# Phenological shifts of low-latitude frogs over 15 years revealed by passive acoustic monitoring

**DOI:** 10.1098/rsos.261532

**Published:** 2026-11-04

**Authors:** Kaede Kimura, Kinji Fukuyama

**Affiliations:** ^1^ Graduate School of Science, Kyoto University, Kyoto, Japan; ^2^ Institute of Agriculture, Tokyo University of Agriculture and Technology, Fuchu, Tokyo, Japan; ^3^ Department of Biology, Keio University, Yokohama, Japan

**Keywords:** climate change, environmental cue, deep learning, bioacoustics, Amphibia

## Abstract

Phenological responses to climate warming are well-documented, yet limited data come from low latitudes. Because phenological sensitivity may vary with latitude, studies in low-latitude regions are essential to understand global patterns. To fill this gap, we analysed acoustic recordings collected daily over 15 years on Iriomote Island, in Japan's transitional zone between subtropical and tropical biomes. We applied the BirdNET deep learning model to quantify the breeding phenology of seven frog species. Seasonal calling patterns differed among species, with peaks occurring in various seasons while one species called nearly year-round. Among six species analysed for phenological trends, a winter-breeding tree-frog (*Zhangixalus owstoni*) showed a statistically significant shift in median calling date (3.8 days later per decade), whereas the other species showed no significant shifts of varying magnitude and direction. Sliding time window analysis indicated late-summer temperature—which increased during the study period—best predicted median calling dates in *Z. owstoni*, suggesting that climate warming delayed the phenology of a winter breeder even where year-round reproduction is possible. The absence of significant phenological advancement in our dataset contrasts with spring advancement commonly reported in temperate regions. These findings reinforce the importance of examining long-term phenological shifts across more sites in low-latitude regions.

## 1. Introduction

Phenological shifts are among the most documented biological responses to recent climate change [1–3]. Although patterns of phenological shifts vary considerably among populations, syntheses across regions reveal an overall trend towards earlier spring phenology at mid- to high latitudes [3,4]. These changes can modify the strength of temporally structured species interactions such as competition, predation and pollination, thereby altering the population dynamics of those interacting species [5,6]. Despite the accumulated reports on phenological shifts, most data come from temperate regions in North America and Europe [3]. Long-term phenological observations remain rare in low-latitude regions, especially for animals [7,8].

The direction and magnitude of phenological shifts among species inhabiting tropical and subtropical regions may differ from those at higher latitudes, since these groups experience seasonality fundamentally differently. For example, lower freezing risks towards the tropics may lessen the importance of temperature for phenology, whereas precipitation and photoperiod may become more important cues, particularly for ectothermic animals and plants [3,9]. Previous meta-analyses of phenological responses suggested that spring phenology is advancing more at higher latitudes [10], and that phenology and temperature may be more tightly related in these regions [3,11], although opposite patterns have also been reported (reviewed in [12]). Importantly, such generalizations of latitudinal clines are based on geographically biased datasets with limited data from areas lower than 30° latitude. Additional tropical and subtropical studies are thus clearly needed to understand global patterns of phenological shifts. However, acquiring the long-term data needed to assess phenological shifts is generally laborious and difficult.

To address a long-standing data limitation and investigate phenological shifts at low latitudes, this study uses a powerful dataset of daily acoustic recordings collected over 15 years from eight sites across Iriomote Island, Japan, as part of a programme originally designed to monitor amphibian population trends. Iriomote Island is in a transitional area between subtropical and tropical zones (24°35′ N), where few long-term phenological data are available. As opposed to the breeding phenology typical of amphibian communities at higher latitudes [13,14], Iriomote's mild winter climate allows some frogs on the island to breed even during the coldest season [15]. Passive acoustic monitoring (PAM) is well suited to continuously monitor breeding phenology of frogs with limited manpower, since breeding males aggregate at waterbodies and call intensively [16]. Until recently, manual species identification has been a major analytical bottleneck. However, emerging deep learning-based automation tools overcome this limitation and enable efficient processing of large acoustic datasets [17].

Here, we use 15-year daily recordings and an automated species classification model to test whether frogs are shifting their breeding phenology on a low-latitude island owing to recent climate warming. Iriomote Island is remote and has very limited data available on frog phenology. As such, we first examine the annual breeding cycles of frogs on Iriomote Island, then assess phenological shifts during the 15-year study period and investigate the climatic cues (temperature and rainfall) that may be driving such shifts. Many meta-analytic studies used phenological observations spanning more than 10 years as an inclusion criterion [2,3,18,19], suggesting that our dataset meets the minimum duration commonly considered suitable for detecting phenological shifts. Nevertheless, 15 years may be insufficient to robustly assess the effects of climate change on amphibian phenology [20]. We therefore place this study as an early contribution to phenological trend observations in low-latitude regions, which provide a foundation for future syntheses assessing how phenological responses to global warming vary across latitudes.

## 2. Material and methods

### 2.1. Acoustic data collection

Our study location was Iriomote Island (24°35′ N, 123°80′ E), Japan, which is 289 km^2^ in size (figure 1a). Iriomote Island has a mild winter climate (18.5°C monthly mean temperature in the coldest month) and is humid throughout the year, with normal monthly precipitation over 100 mm every month (figure 1b). Precipitation peaks during the monsoon-driven rainy season (May–June) and the typhoon season (August–September), followed by a winter period (December–February) with continuous rain and cloudy days (figure 1b). The annual mean temperature on the island has been rising over the past half century at a rate of 0.14°C per decade (figure 1c), while the annual precipitation has no appreciable long-term trend [21]. Our study period spans from 2009 to 2025, during which particularly warm years occurred from 2015 onwards, providing an opportunity to examine amphibian phenological responses to recent temperature fluctuations.

**Figure 1.**
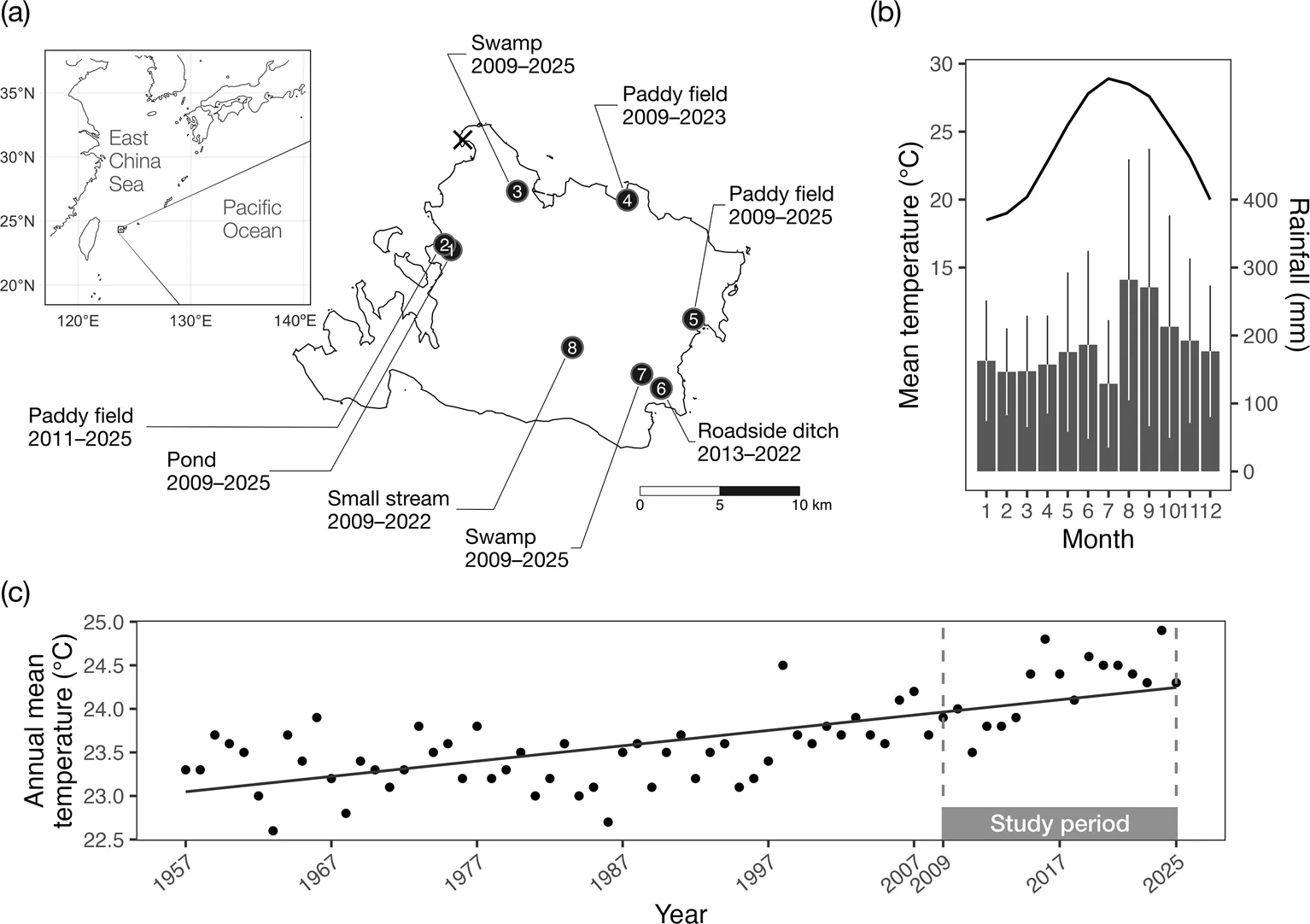
Study sites. (a) Map of recording sites on Iriomote Island, Japan, with a brief description of habitat type and recording years. Cross mark denotes the location of the meteorological station. (b) The climatological normal of monthly mean air temperature and rainfall (± s.d.) at Iriomote Island during 1991–2020. (c) Long-term trend in annual mean temperature showing an increasing trend of 0.14°C per decade after 1957. Dashed lines denote the start and end years of our study period. The map is modified from Digital National Land Information (MLIT, Japan), and the climate data are obtained from the Japan Meteorological Agency.

Iriomote Island hosts eight native frog species, seven of which are endemic to the Sakishima Islands, an archipelago at the southernmost end of Japan. Among them, the two species of *Odorrana* (*Odorrana supranarina* and *Odorrana utsunomiyaorum*) and *Nidirana okinavana* are listed as endangered species [22]. The island was registered as a UNESCO World Heritage Site in 2021.

We deployed autonomous acoustic recorders at eight sites across Iriomote Island between 2009 and 2025 (figure 1a). These sites covered various breeding habitats to record all frog species on this island except for *O. utsunomiyaorum*, whose breeding sites were not discovered until recently (unpublished data). Six of the eight sites were lentic habitats, including a pond of approximately 10 × 20 m, *Barringtonia racemosa* swamp forests and rice paddy fields. In paddy fields, water is filled only during the cultivating seasons (February–June and August–November) [23]. The other pond and swamp sites retained water year-round. We also included a roadside ditch where *Buergeria choui* was abundant (site 6), and a small stream in Mt. Kuwaki where *O. supranarina* breeds (site 8). To make the monitoring system more manageable, we terminated recording at three sites (4, 6 and 8) in 2022. We relocated the recorder at site 7 several hundred metres away the following year, 2023, to a location with audible *O. supranarina* calls.

We used several models of acoustic recorders depending on availability throughout the study period (Sanyo ICR-RSS110M; Olympus DM-720; Panasonic RR-XS series 410, 450, 455, 460, 470). Each recorder was enclosed in a waterproof zipper bag and mesh pipe, and mounted on a tree near the breeding water body at a height of 1–1.5 m [24]. Batteries and SD cards were replaced every several months to a year.

We recorded for 10 minutes per day between 21.00 and 21.10. We chose this recording schedule as the timing is suitable for detecting most frog species in this system, while preventing undue strain on recorder storage and battery [24]. Validation of the recording schedule is provided in supplementary material, S1. Although most species actively vocalize during this period, *Fejervarya sakishimensis* calls mainly around 03.00–05.00, and therefore its activity can be underestimated in our dataset (unpublished data). We recorded at a sampling rate of 44.1 kHz in MP3 format (bit rate 128 or 192 kb s^−1^) for most sites and periods. Exceptionally, recordings were in Windows Media Audio format during March–August 2009 at sites 1, 3 and 8, and at a lower sampling rate (22.05 kHz, 64 kb s^−1^) during December 2011–January 2017 at site 4 and March 2013–January 2017 at site 6. We confirmed that even the lowest sampling frequency and bit rate were suitable to detect the calls of the target taxa.

### 2.2. Training data preparation

To construct an acoustic dataset for model training, we extracted 21–52 audio files from each site (mean = 32). For each file, we used only the first 1 minute. The beginning and ending points of call bouts were annotated in Raven Lite (Cornell Lab of Ornithology), following the protocol of Kimura *et al.* [25]. Segments containing overlapping calls were annotated with multiple species labels. To reduce detection bias across recording settings (sampling rate and bit rate), we annotated at least 20 minutes of audio per setting. We supplemented the dataset with a previously published annotated dataset from the same island and nearby Ishigaki Island [25], yielding a total of 385 minutes of training data. The dataset consisted of one non-frog class (background), seven native frog species (*B. choui, F. sakishimensis, Kurixalus eiffingeri, Microhyla kuramotoi, N. okinavana*, *O. supranarina* and *Zhangixalus owstoni*) and two non-native frog species (*Polypedates leucomystax* and *Rhinella marina*). The annotated recordings were segmented into 3-second clips to fit the model analysis unit. This resulted in 241 (*R. marina*) to 2261 (*M. kuramotoi*) training samples per species (mean = 1088), with some samples containing multiple species.

### 2.3. Automate species classifier development and validation

We used BirdNET v. 2.4 to train a custom classification model [17]. While BirdNET was originally trained mainly on birdsong, it can be applied to other taxa through transfer learning. We trained the model for up to 250 epochs and performed 100 trials of hyperparameter tuning using the ‘autotune’ method in BirdNET. Since BirdNET prevents overfitting by early stopping (i.e. terminating training when model performance no longer improves), we set the maximum number of training epochs sufficiently high based on preliminary trials with different learning rates and data augmentation settings. During hyperparameter tuning, we used 80% of the annotated dataset for training and the remaining 20% for validation [26]. We selected the 0–6 kHz frequency range as most relevant for anuran calls.

BirdNET outputs species classification with a confidence score, ranging from 0 (very low model confidence) to 1 (very high confidence) for each species in each 3-second audio sample. To determine the confidence score beyond which species classifications are accurate, we randomly extracted 200 detections with confidence scores greater than 0.4 for each species from each site and validated them manually by listening to recordings. Based on these validations, we set site- and species-specific confidence thresholds to ensure precision (defined as the proportion of true positives among all positive predictions) exceeded 0.95. We selected a precision value of 0.95 as a practical quality-control criterion to balance high reliability with a sufficient number of detections.

During the validation process, we found calls of the two non-native species (*P. leucomystax* and *R. marina*) at sites 2 and 4. However, these calls lasted only approximately 1 minute, and car sounds were often audible before and after them. These calls were thus probably playbacks used in callback surveys for monitoring alien species on this island. Accordingly, we omitted these non-native species from our analysis.

### 2.4. Characterizing breeding phenology

Since data on the breeding phenology of frogs on Iriomote Island is scarce, we first characterized their breeding cycles. We applied the custom-trained BirdNET model to the whole acoustic recordings (6324 hours). Breeding activity was quantified by the number of daily call detections [27], which ranges from 0 (no detection) to 200 (continuous detection throughout the 10-minute recording) (figure 2).

**Figure 2.**
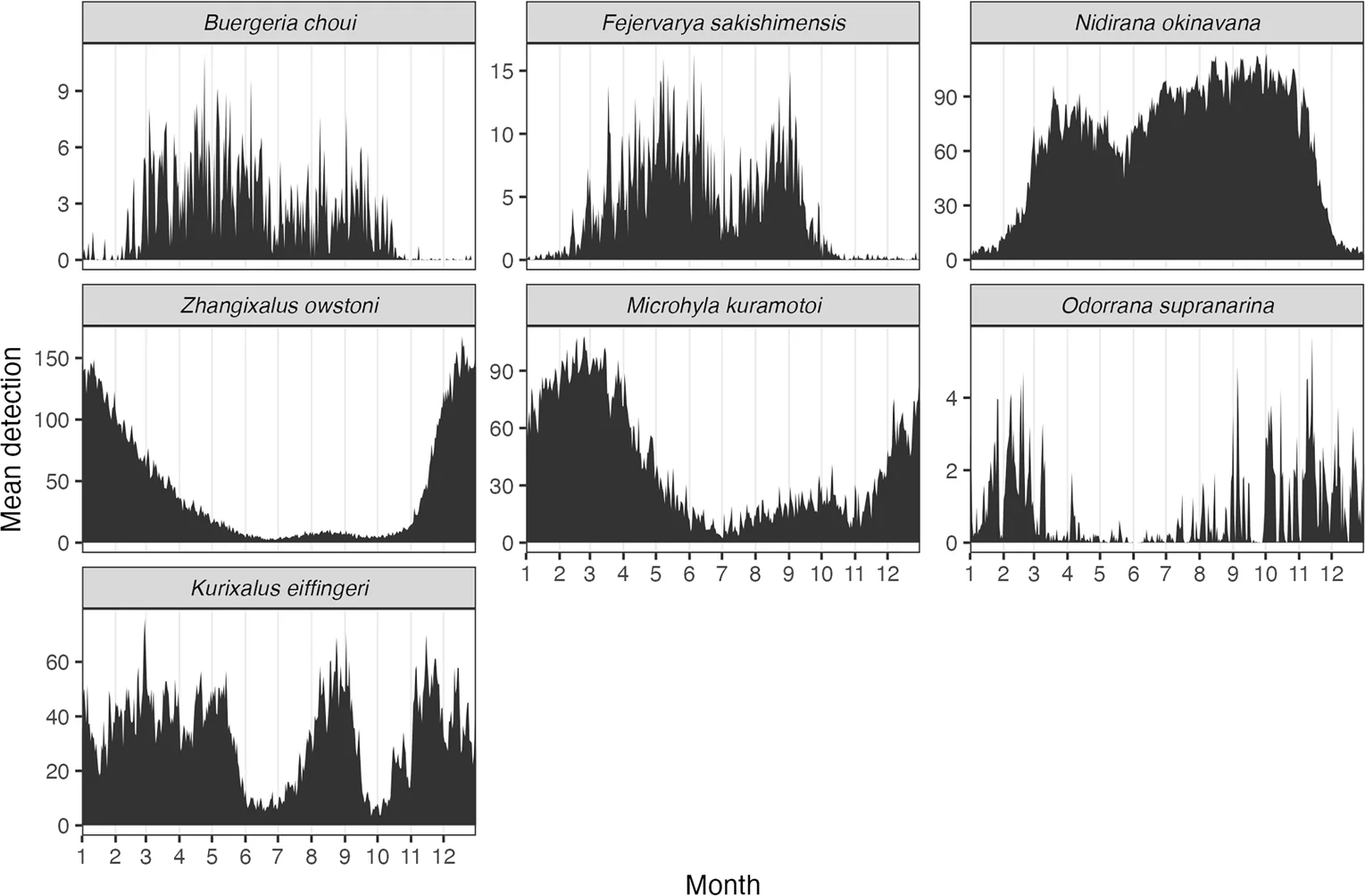
Calling phenology of seven anuran species on Iriomote Island, Japan. The daily number of detections was averaged across years and sites, showing the species’ typical annual cycle. Note that *O. supranarina* is an explosive breeder, but variation in calling dates across years makes this pattern less apparent when data are combined. The number of detections ranges from 0 (no detection) to 200 (continuous detection throughout the 10-minute recording).

### 2.5. Phenological shift assessment

To assess phenological shifts, we defined the *median calling date* as the day of the year on which 50% of the year's total calls had been detected. The median calling date would provide a more robust estimate of species phenology than the first calling date, as it is less sensitive to fluctuations in population size [28]. For species with autumn–spring peak calling activity (*O. supranarina*, *M. kuramotoi* and *Z. owstoni*; see Results), we set the year frame from 1 July of the previous year to 30 June. Among the seven study species, *K. eiffingeri* was unique in having multiple non-breeding seasons (see Results), making it difficult to define median calling dates. We therefore excluded this species from subsequent analysis. To avoid biased estimates of the median calling date, we excluded any site-year with fewer than 500 detections or with any missing recordings confined to only part of the breeding season (owing to recording schedule change, battery depletion or theft).

We tested phenological shifts using a linear mixed model, with median calling date (days since 1 January for species peaking in summer and days since 1 July for species peaking in autumn–spring) as the response variable, numeric year as the fixed effect and site as a random intercept term as follows:

Median calling date∼year+(1 | site).

This model structure reflects our assumption that populations may differ in their median calling dates owing to, for example, microclimate variation, but share a similar linear response to recent climate change, which we consider reasonable given the small size of the island. Regression analyses were conducted in the R environment [29] using the lmerTest package [30].

### 2.6. Phenological cue identification

To identify the climatic cue best explaining variation in median calling dates, we employed a sliding time window approach using the R package climwin [31]. In this approach, we first set a window in calendar dates (e.g. 1–20 March or 10 September–10 December) and calculated the mean of the daily mean temperature (derived from 24-hourly observations), daily minimum temperature and daily precipitation during that window. Each of these climatic variables was then regressed against the median calling date to estimate the effect of climate on the annual breeding cycles. Since our study species are nocturnal, we used daily mean and minimum temperatures rather than daytime maxima as potential phenological cues, following previous studies [32,33]. We set the possible window length to 10–365 days because windows shorter than 10 days are less biologically meaningful and probably represent statistical artefacts. We repeated the model fitting for all possible windows and selected the best model (window) with the largest difference in Akaike's Information Criterion corrected for small samples (ΔAICc) between a baseline model (site effect only) and a model including both climate and site effects. The recording site was treated as a fixed effect in this analysis because random-intercept models were computationally demanding, but this choice should not affect the relative ranking of ΔAICc values among different time windows. Finally, to distinguish real climatic signals from false-positive signals occurring owing to multiple testing, we randomized the median calling dates and repeated the window-search for each randomized dataset to obtain the null distribution of ΔAICc [31]. Randomization was repeated 1000 times. The *p*-value (*P*_∆AICc_) was calculated as the proportion of randomized datasets in which the best-model ΔAICc was lower than the observed ΔAICc.

We obtained daily mean temperature and daily precipitation data from the Iriomote-jima meteorological station of the Japan Meteorological Agency (https://www.data.jma.go.jp/risk/obsdl/index.php). Because missing data were not permitted in the analysis, we imputed two missing precipitation data values as the mean of the preceding and following two days.

## 3. Results

### 3.1. Breeding phenology

We detected calls of *O. supranarina* only at a mountain stream (site 8) and a swamp forest (site 7), and calls of *B. choui* mainly at a roadside ditch (site 6). Other species were widespread and detected at nearly every recording site.

All seven species have long breeding seasons lasting more than six months (figure 2). Among them, three species (*B. choui*, *F. sakishimensis* and *N. okinavana*) have calling peaks in summer. The calling phenology of *B. choui* and *F. sakishimensis* was similar, lasting from February to September and peaking in April–May. *Nidirana okinavana* continued calling later than the other summer species, calling until November with peaks in August–October.

Three species (*Z. owstoni, M. kuramotoi* and *O. supranarina*) peaked during the autumn–spring season (figure 2). *Zhangixalus owstoni* calls peaked in December–January, and *M. kuramotoi* peaked in February. These species were more active in winter but did not completely cease calling in summer. The mountain population (site 8) of *Z. owstoni* remained relatively active even during mid-summer in some years, and *M. kuramotoi* occasionally called intensively on summer nights, though such nights were infrequent compared with the winter–spring season (supplementary material, S2). *Odorrana supranarina* actively called from September to March. Rather than peaking in winter, this species had calling peaks in autumn (September–November) and February, although available data are insufficient to determine whether its breeding season is truly bimodal (figure 2). We also found that *O. supranarina* was an explosive breeder with repeated short breeding events (2–3 days) once every several weeks (supplementary material, S2).

*Kurixalus eiffingeri* reduced calling activity twice a year in June and mid-September to mid-October (figure 2). Such a pattern was repeatedly observed across multiple sites and years (supplementary material, S2).

### 3.2. Long-term trends

Among the six studied species, only *Z. owstoni* significantly shifted its breeding phenology (slope = 0.38, CI: 0.03–0.73, *p* = 0.036; table 1; figure 3). This species was estimated to have delayed its breeding season by 3.8 days per decade during our study period. Three species (*B. choui*, *F. sakishimensis* and *O. supranarina*) exhibited non-significant delays in median calling date (2.5–21.7 days per decade), and two (*N. okinavana* and *M. kuramotoi*) non-significant advances (–0.6 to –5.7 days per decade). Because scheduled artificial irrigation in paddy fields may mask shifts in breeding phenology, we repeated the analysis after excluding the paddy field sites (sites 2, 4 and 5). However, no significant phenological shifts were detected, indicating that the inclusion of paddy field sites did not hinder the detection of phenological shifts.

**Table 1.** Shifts in breeding phenology of six anuran species on Iriomote Island, Japan. Sample size (*N*) represents the number of year-site combinations. Bold text denotes statistical significance.

species	*N*	coefficient	s.e.	*p*
*B. choui*	12	2.17	1.91	0.288
*F. sakishimensis*	50	0.46	1.12	0.685
*N. okinavana*	61	−0.06	1.06	0.958
*Z. owstoni*	91	0.38	0.18	**0.036**
*M. kuramotoi*	80	−0.57	0.64	0.375
*O. supranarina*	11	0.25	2.38	0.920

**Figure 3.**
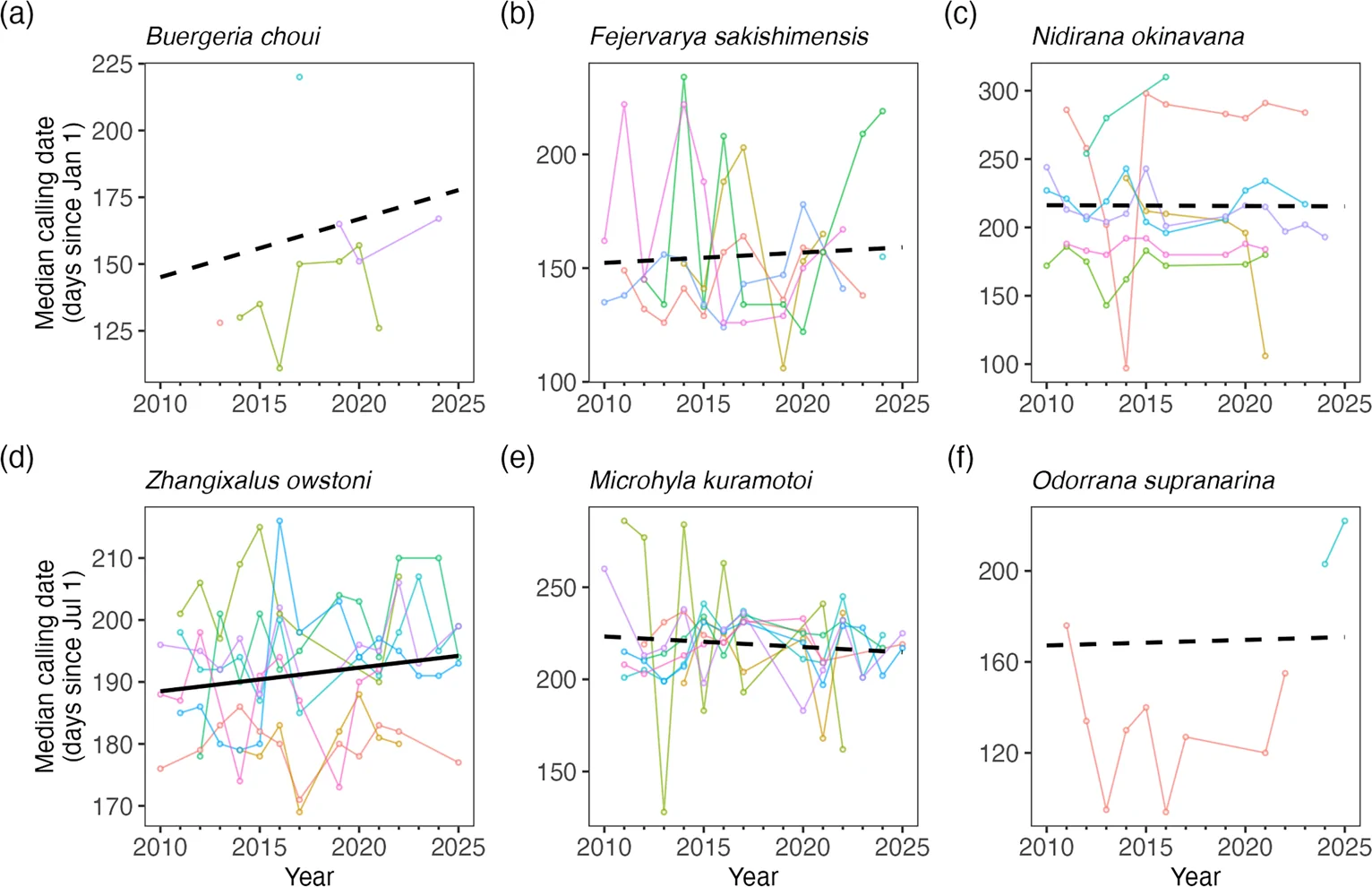
Temporal trends in median calling dates of six anuran species, shown in panels (a–f). Coloured lines connect data from the same population, and black lines represent regression lines averaged across sites (solid: significant trend; dashed: non-significant trend). Only *Z. owstoni* showed a statistically significant trend, with a delay of 3.8 days per decade.

### 3.3. Response to climate

We found evidence of daily minimum temperatures linked to phenology for winter-breeding *Z. owstoni* and summer-breeding *F. sakishimensis* (table 2). Notably, delayed breeding in *Z. owstoni* was correlated with higher mean minimum temperatures from 16 to 26 September (*P*_∆AICc_ = 0.039). Furthermore, temperatures during this time window showed an increasing trend over 2010–2024 (simple linear regression: slope = 0.091, *p* = 0.015; figure 4), suggesting that recent climate warming is linked to the observed phenological delay. For *F. sakishimensis*, earlier median calling dates were correlated with higher minimum temperatures from 18 February to 22 March (*P*_∆AICc_ = 0.018), indicating that warmer spring advances the breeding timing in this species (table 2; supplementary material S3). By contrast to *Z. owstoni*, we found no evidence of temporal trends in the climatic cues relevant to *F. sakishimensis* (slope = –0.005, *p* = 0.912). For these species, the responses to daily mean temperature were similar to those to daily minimum temperature (table 2), but daily minimum temperature provided a better fit (*Z. owstoni*: ∆AICc = –10.11 versus –8.42; *F. sakishimensis*: ∆AICc = –10.81 versus –8.86).

**Table 2.** Best climate windows explaining the variation in median calling date estimated by the sliding time window analysis. Bold text denotes statistical significance.

species	climate variable	best window	coefficient^1^	s.e.	$P_{\Delta \text{AI}C_{c}}$
*B. choui*	mean temp.	12 Jan–3 Feb	28.00	5.84	0.195
	minimum temp.	13 Jan–7 Feb	20.52	5.32	0.449
	rainfall	5 Feb–5 Aug	9.33	3.24	0.817
*F. sakishimensis*	mean temp.	16 Feb–22 May	–16.33	4.85	0.073
	**minimum temp.**	18 Feb–22 May	–15.26	4.15	**0.018**
	rainfall	21 Jan–19 Apr	–7.85	2.36	0.127
*N. okinavana*	mean temp.	12 Nov–22 Nov	5.39	2.83	0.293
	minimum temp.	2 Nov–6 Dec	8.58	4.29	0.255
	rainfall	22 Aug–10 Sep	1.90	0.80	0.088
*Z. owstoni*	mean temp.	24 Aug–21 Sep	4.86	1.50	0.100
	**minimum temp.**	16 Sep–26 Sep	3.97	1.14	**0.039**
	rainfall	29 Jan–8 Feb	–1.12	0.36	0.163
*M. kuramotoi*	mean temp.	6 Aug–16 Aug	–25.58	10.34	0.319
	minimum temp.	22 Jun–2 Jul	7.52	2.97	0.299
	rainfall	21 Jun–1 Jul	–3.05	1.28	0.440
*O. supranarina*	mean temp.	3 Aug–16 Aug	–78.47	20.43	0.559
	minimum temp.	6 Jan–16 Jan	–10.32	3.28	0.815
	rainfall	11 Jul–20 Aug	8.83	2.35	0.851

^1^ Shift in median calling date per unit change in climate variable (d °C^–1^ or d mm^–1^).

**Figure 4.**
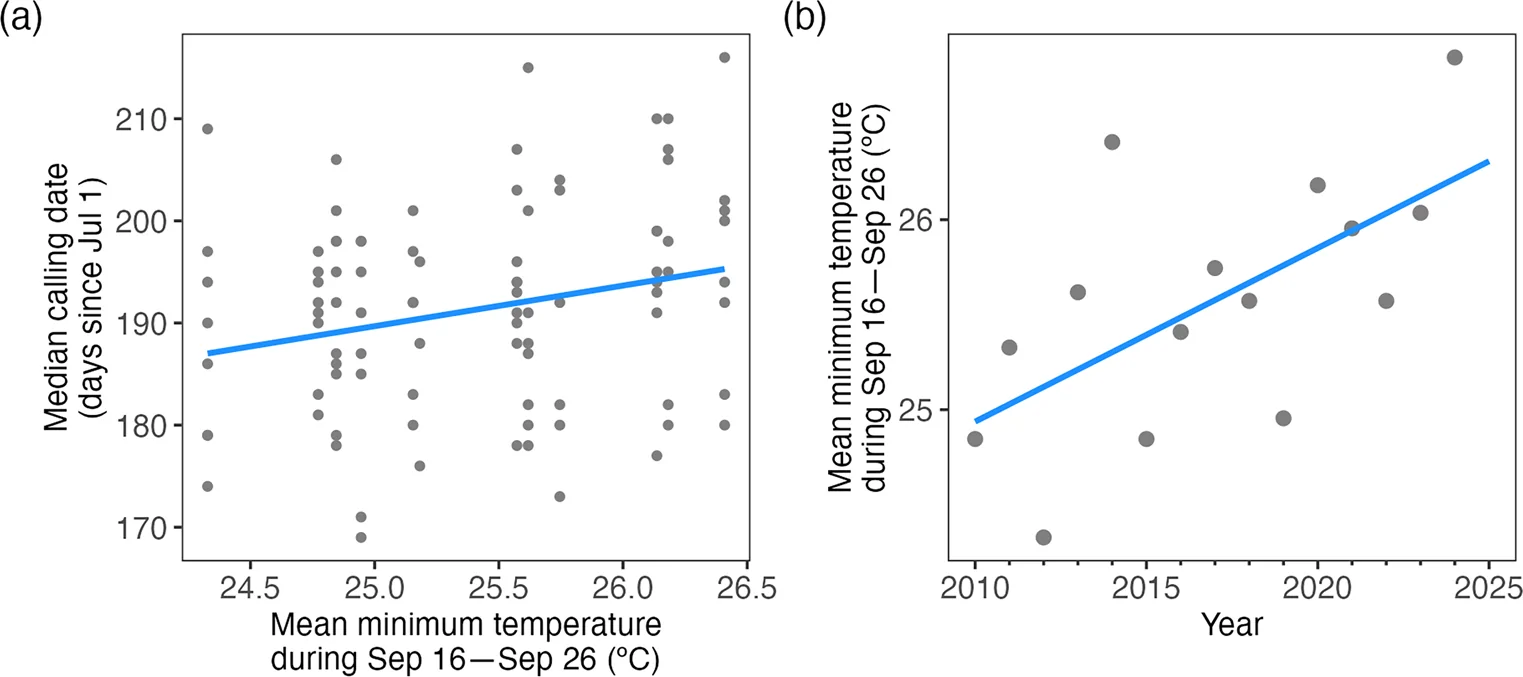
The best climatic cue of *Z. owstoni* breeding phenology. (a) Relationships between the best climatic cue (mean minimum temperature during 16–26 September) and median calling dates of *Z. owstoni*. (b) An increasing trend in the climatic cue during the study period.

## 4. Discussion

Here, we documented the breeding phenology of seven frog species inhabiting Iriomote Island and analysed phenological shifts over 15 years. To our knowledge, this is the first long-term monitoring of detailed amphibian breeding activities in the subtropics. By employing PAM and emerging machine learning tools, we addressed a knowledge gap in the study of phenological shifts in response to global climate warming. In addition, long-term breeding data are essential to understand changes in amphibian population status [34]; our automated acoustic monitoring approach should also contribute to the conservation of frog species.

Although most species showed no significant phenological shifts, *Z. owstoni*, a winter-breeding tree-frog, delayed its breeding season by 3.8 days per decade. The sliding time window analysis suggested that this delay was associated with increasing minimum temperatures in late summer. As a winter-breeding species, *Z. owstoni* may be adapted to reproduce under relatively cool conditions and therefore breed later in warmer years. Such response is consistent with findings of Todd *et al.* [33], who reported delayed responses to warming local climates in autumn-breeding amphibians of South Carolina, USA. Our results demonstrate that even at low latitudes where year-round reproduction is possible, recent climate warming may be changing the phenology of a frog species.

No evidence of advancing breeding phenology was found in frogs on Iriomote Island. This pattern contrasts with studies on animal phenology from temperate regions, which typically report advances in spring phenology, such as shifts by –2.9 days per decade across animals [3] or by –5.3 days per decade for amphibians in particular (based on [11], but we added the two autumn breeders excluded in the original article). This discrepancy may reflect that few amphibians on Iriomote Island are highly responsive to spring temperature. In temperate amphibians, much of the evidence for phenological advancement derives from early-spring breeders that aggregate soon after the end of winter and breed within a short period [32,35–37]. Such species are expected to be sensitive to local temperatures because of freezing risk [38]. However, the breeding phenology on Iriomote Island was characterized by prolonged and diverse breeding seasons with peaks occurring either in summer, from autumn to spring, or nearly year-round. Similarly, breeding seasons in other low-latitude regions are typically long or coincide with the wet season rather than with spring [39–43]. Many low-latitude amphibians may therefore use different cues to initiate breeding than temperate spring breeders and may exhibit phenological responses other than spring advancement as in *Z. owstoni*. Although only *F. sakishimensis* showed earlier breeding in warmer springs, the spring temperature cue associated with its breeding did not increase during the study period, preventing detectable phenological advances. Since our analysis is limited to six amphibian species, additional long-term phenological studies from tropical and subtropical regions are needed to evaluate how phenological responses vary across latitudes. We should note that the 15-year duration of our study may have been insufficient to detect phenological advancement, and our analyses were limited to shifts in median calling dates. Future studies should evaluate a broader range of phenophases (e.g. breeding onset and duration), while accounting for population size fluctuations [28], and should extend observations over longer time periods.

We found evidence that temperature influenced median calling dates in two species. Winter-breeding *Z. owstoni* responded to late-summer temperatures, and summer-breeding *F. sakishimensis* responded to spring temperatures. For these species, daily minimum temperature better predicted median calling dates than daily mean temperature, possibly reflecting the nocturnal activity of frogs. Although several studies have suggested that temperature becomes a less important phenological cue towards the tropics [3,11], seasonal variation of temperature on Iriomote Island may be sufficient to provide an important cue for breeding. We failed to identify phenological cues for many species probably because the sliding time window analysis requires a large sample size (number of different sites or years) when the effect of a climate variable on phenology is not strong [31]. A simulation study reported that when the effect sizes are moderate (*R*^2^ = 0.2), the type II error rate remains approximately 0.1 with nearly 50 samples, indicating the difficulty of convincingly identifying climatic cues [31]. To better understand the environmental drivers of breeding phenology in low-latitude regions, where amphibians might be less responsive to temperature [11], monitoring over longer time periods and across a broader range of locations will be required.

Although a phenological shift was detected in *Z. owstoni*, its consequences for population or community dynamics remain unknown. Even a small difference (less than a week) in the timing of tadpole hatching can influence competitive outcomes between species [44–48]. Therefore, the estimated 5.8-day call date delay over the 15 years in *Z. owstoni* may have implications for species interactions. Among the anurans on Iriomote Island, *N. okinavana* most likely competes with *Z. owstoni* because both exhibit similar lentic-benthic larval forms (personal observations). Their clearly reversed breeding phenology may further indicate a competitive relationship (figure 2). If these species compete strongly, the delayed breeding of *Z. owstoni* could expose its larvae to stronger competitive pressure from more developed *N. okinavana* larvae. Alternatively, since their peak breeding seasons were originally separated by two to three months, the actual impact of this delay might be limited. Predicting the impacts of multi-species phenological shifts is more challenging because competitive outcomes can be species-specific and depend on environmental resource levels [47,48]. Experimental studies are therefore needed to better understand the population- and community-level impacts of phenological shifts in future.

We shed new light on the breeding phenology of Iriomote frogs. All studied species showed long breeding seasons (greater than 6 months), yet individual species did not call consistently year-round, rather having clear annual cycles. Such patterns may reflect temporal niche separation among the potentially competing species and a mild climate on Iriomote Island, which lacks severe winters or dry seasons. Several species also exhibit particularly unusual phenology. *Kurixalus eiffingeri* decreased calling twice a year in June and mid-September to mid-October, and *O. supranarina* exhibited multiple explosive-breeding events within a year. Investigating the adaptive significance of such intriguing phenology is beyond the scope of this study. However, it should be an important avenue of future research to understand how adaptation to low-latitude environments shapes the diverse phenology of each species, and how it relates to interspecific variation in phenological shifts [49].

This study also highlighted the potential of long-term acoustic data in combination with deep learning for identifying phenological shifts in remote locations. In total, we analysed 6342 hours of acoustic recordings, which yielded daily-resolution multi-species phenology data—an outcome made possible through the use of an automated species classification model. Long-term phenological observations are laborious, and hence previous studies often rely on opportunistic observations [50,51] or have a narrower scope in their number of field sites and species [32]. By contrast, PAM enables systematic phenological observations in a cost- and labour-efficient way. With the development of deep learning-based species identification methods, analysing the enormous amount of data from extended deployments has become increasingly feasible [52]. This approach should be particularly effective for monitoring bird migration, anuran calling and insect singing [53]. These methods have several drawbacks, including some false-positives/false-negatives and the need to manually create training datasets for species that the original BirdNET model was not trained for. Additionally, abundance estimation is often difficult solely from PAM data [54], making it challenging to investigate the effects of phenological shifts on population dynamics. Despite these limitations, PAM and deep learning have great potential to broaden our understanding of phenological shifts in previously understudied taxa and regions.

## 5. Conclusion

We presented detailed information on the breeding phenology of a low-latitude frog community over 15 years and found varying phenological trends among species, while also detecting delayed breeding in one species, probably owing to recent climate warming. This is the lowest-latitude study reporting shifts in amphibian breeding phenology to date [1,11], and one of only a few examples of delayed autumn–winter breeding in amphibians [33]. Unlike in temperate zones, where spring breeding typically advances, no species exhibited significant advancement in this study. This case study implies species inhabiting regions without severe winters such as Iriomote Island might exhibit different phenological shifts from those in temperate regions, although substantially more phenological datasets from tropical and subtropical regions are needed to test this possibility. As PAM gains popularity as an ecosystem monitoring tool, acoustic monitoring networks are increasingly being established worldwide (e.g. [55–57]). Continued operation of these initiatives, together with the development of species-recognition algorithms covering a broader range of taxa, could provide insights into how and why phenological shifts vary across latitudes.

## Supplementary Material



## Data Availability

The data and code that support the findings of this study are available in Zenodo [58]. Supplementary material is available online [59].
