# Supplementary material for: Phenological shifts of low-latitude frogs over 15 years revealed by passive acoustic monitoring
Source: R Soc Open Sci. 2026 Nov 4;13(11):261532. doi: 10.1098/rsos.261532 (PMC13730656; doi:10.1098/rsos.261532)
Supplement: Supplementary file 1 [file rsos.261532.zip › rsos.261532/rsos261532_si_001.docx]

**Supplementary material 1 for:
Phenological shifts of low-latitude frogs over 15 years revealed by passive acoustic monitoring**

Kaede Kimura^1,2,*^ and Kinji Fukuyama^3^

Published in *Royal Society Open Science* (doi: 10.1098/rsos.261532)

^1^Graduate School of Science, Kyoto University, Kyoto, Japan

^2^Institute of Agriculture, Tokyo University of Agriculture and Technology, Tokyo, Japan

^3^Department of Biology, Keio University, Yokohama, Kanagawa, Japan

^*^Corresponding author: kaede.kmr@gmail.com

ORCID ID (K.K.): 0009-0001-4009-1424

**Validation of recording schedule**

Using a separate acoustic dataset collected for one minute at hourly intervals (totaling 24 recordings per day) at site 1, we confirmed that recordings at 21:00 adequately captured seasonal variation in calling activity for all species except *Buergeria choui* and *Odorrana supranarina*, which rarely called at this site (Figure S1). Based on these results, we consider our recording schedule did not substantially bias estimates of calling phenology at least for these five species. The data also revealed a low detection rate of *Fejervarya sakishimensis* around 21:00 (this species is more active before dawn).

**Figure S1**. Validation of the recording schedule. A dataset recorded at hourly intervals over an year was used to assess whether call detections at 9 PM (a) were consistent with those obtained from all-day data (b).
