# Supplementary material for: Phenological shifts of low-latitude frogs over 15 years revealed by passive acoustic monitoring
Source: R Soc Open Sci. 2026 Nov 4;13(11):261532. doi: 10.1098/rsos.261532 (PMC13730656; doi:10.1098/rsos.261532)
Supplement: Supplementary file 1 [file rsos.261532.zip › rsos.261532/rsos261532_si_002.docx]

**Supplementary material 2 for:
Phenological shifts of low-latitude frogs over 15 years revealed by passive acoustic monitoring**

Kaede Kimura^1,2,*^ and Kinji Fukuyama^3^

Published in *Royal Society Open Science* (doi: 10.1098/rsos.261532)

^1^Graduate School of Science, Kyoto University, Kyoto, Japan

^2^Institute of Agriculture, Tokyo University of Agriculture and Technology, Tokyo, Japan

^3^Department of Biology, Keio University, Yokohama, Kanagawa, Japan

^*^Corresponding author: kaede.kmr@gmail.com

ORCID ID (K.K.): 0009-0001-4009-1424

This supplementary material contains seven figures showing the number of call detections over time for seven frog species on Iriomote Island from 2009 to 2025.

**Figure S1** Calling phenology of *Buergeria choui* at 8 sites on Iriomote Island, Japan, inferred from the custom trained BirdNET model (year: 2009 to 2017). Grey areas indicate missing data. Number of detections ranges from 0 (no detection) to 200 (continuous detection throughout the 10-min recording). Note that detections may contain false positives.

**Figure S1** Continued (year: 2018 to 2025).

**Figure S2** Calling phenology of *Fejervarya sakishimensis*. Other captions are same as Figure S1.

**Figure S2** Continued.

**Figure S3** Calling phenology of *Nidirana okinavana.* Other captions are same as Figure S1.

**Figure S3** Continued.

**Figure S4** Calling phenology of *Zhangixalus owstoni.* Other captions are same as Figure S1.

**Figure S4** Continued.

**Figure S5** Calling phenology of *Odorrana supranarina.* Other captions are same as Figure S1.

**Figure S5** Continued.

**Figure S6** Calling phenology of *Micohyla kuramotoi.* Other captions are same as Figure S1.

**Figure S6** Continued.

**Figure S7** Calling phenology of *Kurixalus eiffingeri.* Other captions are same as Figure S1.

**Figure S7** Continued.
