# Supplementary material for: Phenological shifts of low-latitude frogs over 15 years revealed by passive acoustic monitoring
Source: R Soc Open Sci. 2026 Nov 4;13(11):261532. doi: 10.1098/rsos.261532 (PMC13730656; doi:10.1098/rsos.261532)
Supplement: Supplementary file 1 [file rsos.261532.zip › rsos.261532/rsos261532_si_003.docx]

**Supplementary material 3 for:
Phenological shifts of low-latitude frogs over 15 years revealed by passive acoustic monitoring**

Kaede Kimura^1,2,*^ and Kinji Fukuyama^3^

Published in *Royal Society Open Science* (doi: 10.1098/rsos.261532)

^1^Graduate School of Science, Kyoto University, Kyoto, Japan

^2^Institute of Agriculture, Tokyo University of Agriculture and Technology, Tokyo, Japan

^3^Department of Biology, Keio University, Yokohama, Kanagawa, Japan

^*^Corresponding author: kaede.kmr@gmail.com

ORCID ID (K.K.): 0009-0001-4009-1424

**Estimated climatic cue for *Fejervarya sakishimensis***

**Figure S1** The best climatic cue of *Fejervarya sakishimensis* breeding phenology. (a) Relationships between the best climatic cue (mean minimum temperature during February 18 to March 22) and the median calling dates. Higher spring temperatures advance the breeding phenology. (b) No evidence of a temporal trend in the climatic cue over the study period (slope = -0.005, *p* = 0.912).
